# Cognitive function among hemodialysis patients in Japan

**DOI:** 10.1186/1744-859X-10-20

**Published:** 2011-08-25

**Authors:** Gen Odagiri, Norio Sugawara, Atsuhiro Kikuchi, Ippei Takahashi, Takashi Umeda, Hisao Saitoh, Norio Yasui-Furukori, Sunao Kaneko

**Affiliations:** 1 Department of Neuropsychiatry, Hirosaki University School of Medicine, Hirosaki, Japan; 2 Department of Social Medicine, Hirosaki University School of Medicine, Hirosaki, Japan; 3 Department of Urology, Oyokyo Kidney Research Institute, Hirosaki Hospital, Hirosaki, Japan

## Abstract

**Background:**

Over 290,000 patients are undergoing hemodialysis (HD) in Japan. With old age, the odds of undergoing HD treatment sharply increase, as does the prevalence of cognitive impairment. The aim of the present work was to assess cognitive impairment in HD patients and its relation to clinical characteristics.

**Methods:**

Using a cross-sectional design, we administered the Mini-Mental State Examination (MMSE) to 154 HD outpatients and 852 participants from the Iwaki Health Promotion Project 2010, representing the general population.

**Results:**

The prevalence of cognitive impairment based on the MMSE was 18.8% in HD patients. HD patients showed a higher prevalence of cognitive impairment in older groups (50 years and older). In a logistic regression model with age, gender and amount of education as covariates, undergoing HD was a significant independent factor (OR = 2.28, 95% CI 1.33 to 3.94) associated with a lower MMSE score. Among HD patients, we found that level of education was associated with MMSE score.

**Conclusions:**

There is a high prevalence of cognitive impairment among HD patients that has adverse implications for hospitalization and shortens their life expectancy. HD treatment was an independent risk factor for cognitive impairment. Clinicians should carefully monitor and treat cognitive impairment in HD patients. Further studies are required to determine the reasons for cognitive impairment in HD patients.

## Introduction

Advances in medical technology and improvements in public health have brought about a progressive increase in the population undergoing hemodialysis (HD). Over 290,000 patients receive HD in Japan, which has the world's highest rate of dialysis treatment (2,280 per million people) as of December 2009 [1]. The prevalence of HD treatment sharply increases in old age, as does the prevalence of cognitive impairment.

Recently, the relationship between HD and cognitive impairment has attracted attention [2-5], and its causes have been discussed. A previous study [3] of 80 HD patients (mean age, 61.2 ± 14.3 years) found severe levels of impairment in executive function (38%), as measured by the Trail Making Test B, Part B (Trails B), and severe memory impairment (33%) based on the short form of the California Verbal Learning Trial (CVLT). A more recent study [4] assessing cognitive function across multiple cognitive domains in 338 HD patients (mean age, 71.2 ± 9.5 years) showed that the 37% of patients had severe cognitive impairment. In older HD patients, a French study [6] showed that the prevalence of cognitive impairment based on the Mini-Mental State Examination (MMSE) was 47% of 51 HD outpatients (at least 70 years old).

The etiology of cognitive impairment among HD patients is thought to be multifactorial, and includes factors such as cerebrovascular lesion [7,8], hypotension [9,10], abnormalities of serological data [2,11], social history [12] and amount of HD [3,13]. In addition, the high prevalence of cardiovascular risk factors might overshadow the roles of aging and non-vascular factors in the development of cognitive impairment [14-16].

There have only been a few studies [3,4,17] that compared the features of cognitive function between HD patients and the non-HD population. It is therefore necessary to accurately assess the features of cognitive function cross-sectionally, especially in comparison with a healthy reference group.

In this study, we assessed the risk factors for cognitive impairment in HD patients and investigated the prevalence of cognitive impairment among HD patients compared with that of the general population in Japan. To the best of our knowledge, this is the largest study to date to assess the effect of HD on cognitive function and the first report comparing HD patients with the general population in Japan.

## Methods

### Participants

This study was conducted between September 2009 and January 2010. A total of 154 patients (88 males and 66 females) undergoing HD were recruited at Oyokyo Kidney Research Institute in Japan. Demographic data (age, gender, amount of education) were obtained from self-questionnaires and interviews. Clinical information (duration of hemodialysis, dry weight) was obtained from medical charts. Blood sampling was performed no later than 2 weeks prior to cognitive assessment. Red blood cell count, albumin, sodium, potassium, uric acid, creatinine, blood urea nitrogen and amount of hemodialysis were also measured by standard analytical techniques. As a reference group, 852 healthy volunteers (314 males and 538 females, aged 30 years and above) who participated in the Iwaki Health Promotion Project 2008 were also included. The data collection for this study was approved by the Ethics Committee of the Hirosaki University School of Medicine and all subjects provided written informed consent before participating in this study.

### Assessment of cognitive impairment

The MMSE [18] was given to all participants to measure their global cognitive status. This test assesses orientation to place and time, short-term memory, episodic long-term memory, subtraction, ability to construct a sentence and oral language ability. The maximum score was set as 30 and poor cognition was defined as a score of less than 24 [19].

### Statistical analysis

Data are presented as mean ± SD. A value of *P *<0.05 was considered significant. The unpaired Student's t test was performed to analyze continuous variables, and a χ^2 ^test or Fisher's exact test was performed to analyze categorical variables. Although we divided the subjects into age subgroups by decades, the age-specific prevalence of a lower MMSE score was analyzed across three larger groups (30-49, 50-69 and 70+ years old) due to the small sample size of each smaller subgroup. To assess the relationship between undergoing HD and poor cognitive function, a logistic regression analysis was performed after adjusting for confounding factors (age, gender and amount of education). In addition, a logistic regression analysis was also applied to determine the factors associated with MMSE score among the HD patient group. The data were analyzed using the PASW Statistics software (version 18.0.0) for Windows (SPSS Inc., Chicago, IL, USA).

## Results

### Clinical and demographic characteristics of the subjects

The prevalence of cognitive impairment based on the MMSE score was 18.8% among HD patients and 6.0% among control. Table 1 shows the clinical and demographic characteristics among the subjects. Compared with the controls, the HD patients were older, had less education and had lower MMSE scores.

**Table 1:** Clinical and demographic characteristics of study subjects

	Patients on hemodialysis n = 154	Controls n = 852	*P *value
Age	65.1 ± 13.3	57.8 ± 12.2	0.001
Gender	M 88, F 66	M 314, F 538	0.001
Duration of education (years)	10.7 ± 2.5	11.3 ± 2.1	0.01
MMSE score	26.6 ± 3.9	28.1 ± 2.4	0.001
Duration of hemodialysis (years)	7.8 ± 6.4		
Erythrocyte count (10^4^/mm^3^)	353.0 ± 41.2		
Albumin (g/dl)	3.7 ± 0.4		
Sodium (mEq/l)	139.1 ± 3.4		
Potassium (mEq/l)	4.8 ± 0.8		
Uric acid (mg/dl)	7.6 ± 1.4		
Creatinine (mg/dl)	10.7 ± 3.2		
Blood urea nitrogen (mg/dl)	62.1 ± 15.8		
Dry weight (kg)	55.1 ± 13.9		
Amount of hemodialysis (Kt/V)	1.83 ± 4.50		

Data are presented as mean ± SD.

*Indicates a significant difference (*P *< 0.05) between groups.

MMSE = Mini-Mental State Examination.

### Risk factors associated with having lower MMSE scores (<24)

Table 2 shows the age-specific prevalence of lower MMSE (<24) among patients with HD and the general population in Japan. HD patients showed a higher prevalence in the older groups (50-69, 70+ years old) but not in the youngest group (30-49 years old). The effect of gender and education on prevalence of lower MMSE (<24) is shown in Tables 3 and 4, respectively. In a logistic regression model with age gender and amount of education as covariates (Table 5), undergoing HD was a significant independent factor (odds ratio = 2.28) having poor cognitive function.

**Table 2:** Age-specific prevalence of lower Mini-Mental State Examination (MMSE) scores (<24) in patients with hemodialysis and the general population in Japan

Age group	Patients on hemodialysis		Controls	
	% ± SD	n/N	% ± SD	n/N
30-39 years old	12.5 ± 11.7	1/8	1.3 ± 1.3	1/75
40-49 years old	0.0 ± 0.0	0/14	0.7 ± 0.7	1/144
50-59 years old	8.7 ± 5.9	2/23	2.5 ± 1.0	6/239
60-69 years old	16.3 ± 5.3	8/49	8.7 ± 1.9	20/229
70-79 years old	29.3 ± 7.1	12/41	13.6 ± 2.8	21/154
80+ years old	31.6 ± 10.7	6/19	18.2 ± 11.6	2/11

**Table 3:** Gender-specific prevalence (%) of lower MMSE scores (<24)

Sex	Patients on hemodialysis		Controls	
	**% **± **SD**	n/N	**% **± **SD**	n/N
Male*	15.9 ± 3.9	14/88	8.0 ± 1.5	25/314
Female*	22.7 ± 5.2	15/66	4.8 ± 0.9	26/538

The obtained data were analyzed using a χ^2 ^test or Fisher's exact test between HD patients and controls.

*Indicates a significant difference (*P *<0.05) between groups.

**Table 4:** Prevalence (%) of lower Mini-Mental State Examination (MMSE) scores (<24) among participants segregated according to educational level

Age group	Patients on hemodialysis		Controls	
	% ± SD	n/N	% ± SD	n/N
1-9 years*	29.2 ± 5.6	19/65	14.3 ± 2.1	38/266
10-12 years*	13.9 ± 4.1	10/72	2.6 ± 0.7	12/460
13+ years	0 ± 0	0/17	0.7 ± 0.7	1/147

The obtained data were analyzed using a χ^2 ^test or Fisher's exact test between HD patients and controls.

*Indicates a significant difference (*P *<0.05) between groups.

**Table 5:** Risk factors associated with having lower Mini-Mental State Examination (MMSE) scores (<24) estimated by logistic regression analysis

Independent variables	Odds ratio	95% CI	*P *value
Age	1.05	1.02 to 1.08	0.01
Gender	1.52	0.93 to 2.50	0.098
Duration of education (years)	0.76	0.66 to 0.86	0.001
Hemodialysis	2.28	1.33 to 3.94	0.01

The multiple logistic regression model included all of the above-mentioned factors as independent variables.

### Factors that influenced the MMSE scores among HD patients

The results of a logistic regression model that included age, gender, amount of education, duration of hemodialysis, red blood cell count, albumin, sodium, potassium, uric acid, creatinine, blood urea nitrogen, dry weight, amount of hemodialysis, and comorbidities are shown in Table 6. Amount of education was independently and significantly associated with the MMSE score. Serum sodium concentration, dry weight and having cerebrovascular disease approached statistical significance (*P *< 0.10).

**Table 6:** Risk factors associated with having lower Mini-Mental State Examination (MMSE) scores (<24) estimated by logistic regression analysis

Independent variables	Odds ratio	95% CI	*P *value
Age	1.01	0.95 to 1.06	0.830
Gender	1.42	0.36 to 5.56	0.619
Amount of education (years)	0.74	0.57 to 0.97	0.05
Duration of hemodialysis (years)	1.03	0.93 to 1.13	0.608
Erythrocyte count (10^4^/mm^3^)	1.00	0.98 to 1.01	0.537
Albumin (g/dl)	1.54	0.37 to 6.37	0.549
Sodium (mEq/l)	0.86	0.74 to 1.00	0.05
Potassium (mEq/l)	0.77	0.33 to 1.83	0.558
Uric acid (mg/dl)	0.79	0.52 to 1.20	0.265
Creatinine (mg/dl)	0.95	0.75 to 1.21	0.681
Blood urea nitrogen (mg/dl)	1.01	0.97 to 1.05	0.578
Dry weight (kg)	0.94	0.88 to 1.00	0.061
Amount of hemodialysis (Kt/V)	0.27	0.06 to 1.36	0.113
History of diabetes mellitus	2.07	0.66 to 6.46	0.211
History of hypertension	2.04	0.76 to 5.48	0.157
History of heart disease	1.16	0.34 to 4.02	0.810
History of cerebrovascular disease	3.07	0.98 to 9.66	0.055

The multiple logistic regression model included all above-mentioned factors as independent variables.

## Discussion

In Japan, the mean age of HD patients has changed considerably over time, from 48.3 years old in 1983 to 65.8 years old in 2009 [1]. Aging is associated with cognitive impairment, which can cause various behavioral and psychological symptoms [20]. In patients undergoing HD, cognitive impairment brings more serious consequences, such as hospitalization and reduced life expectancy [11,21]. Cognitive impairment in HD patients might hinder them from complying with dialysis schedules, medications, and dietary restrictions.

In this study, 18.8% of HD patients were classified as having cognitive impairment. Among those who were 50 years or older, the prevalence of cognitive impairment was higher among HD patients than among the controls. We also found that HD patients had higher risk for poor cognitive function even after adjusting for covariates. Among HD patients, level of education was associated with MMSE score. In addition, serum sodium level, dry weight and history of cerebrovascular disease tended to associate with MMSE score of HD patients.

Table 2 shows the age-specific prevalence of lower MMSE scores (<24) in HD patients and in the control group. First, we divided the subjects into age subgroups by decade. However, these groups were then integrated into three larger groups (30-49, 50-69 and at least 70 years old) to analyze the effect of aging because of the small sample size, which caused wide variability in the performance of each subgroup. There was little difference among the younger groups. However, in the older age group, the HD patients had a higher prevalence of cognitive impairment than did the controls. A possible explanation is that the failure to find a difference in the prevalence of cognitive impairment in those aged 49 years and younger is due to the smaller sample size of this group. There might be insufficient power to detect associations within such a small group. Therefore we could not rule out the possibility of beta errors affecting our results.

The logistic regression analysis of risk factors associated with lower MMSE scores is shown in Table 5. After adjusting for covariates, HD treatment (OR = 2.28, 95% CI 1.33 to 3.94) was shown to be an independent risk factor. Murray *et al. *[5] showed a higher risk (odds ratio = 3.54) of having severe cognitive impairment among HD patients compared to non-HD controls. They assessed cognitive function across three cognitive domains: memory, executive function, and language, using nine validated neuropsychological tests. Their odds ratio was higher than that of our study, possibly due to differences in the neuropsychological tests used. Our results do not mean that HD treatment itself is a risk factor for cognitive impairment, because the duration of HD does not have significant relationship to MMSE scores. A previous study [22] showed that lower estimated glomerular filtration rate (eGFR) relates to lower cognitive function in chronic kidney disease (CKD) patients. Cognitive impairment of HD patients might be due to CKD prior to kidney failure.

Previous studies have shown a relationship between cerebrovascular disease and cognitive impairment [23,24]. The prevalence of stroke in the United States Renal Data System (USRDS) HD population is 17%, compared with 4% in the general Medicare population [25]. The proportion experiencing a stroke each year is almost as high; the incidence is 15% for HD patients and 2.4% for the non-chronic kidney disease population. Stroke is also 6-9 times more common in hospitalized HD patients than in non-HD patients [26]. HD patients without cerebrovascular disease in Japan had a lower prevalence of dementia (7.2%) than did HD patients with cerebrovascular disease (23.1%) [1]. In this study, the incidence having lower MMSE scores (<24) is 34.6% (9/26) for HD patients with cerebrovascular disease and 15.6% (20/128) for HD patients without cerebrovascular disease population. Although cerebrovascular disease might explain a part of the cognitive impairment among HD patients, it could not fully explain all causes of their impairment.

We also found a relationship (*P *= 0.05) between hyponatremia and the MMSE score among HD patients. This relationship was also reported in a previous study by Maugeri *et al. *[14]. Hyponatremia depends on various factors including blood dilution by chronic fluid overload and dietary sodium restriction [27]. These factors might explain why no correlations were observed with other blood parameters, which are stabilized by HD to some extent.

There are several limitations to our study. First, we administered only the MMSE for assessing the cognitive function. Although the MMSE is suitable for screening of some cognitive functions including orientation to place and time, short-term memory, episodic long-term memory, subtraction and attention, the MMSE score does not always reflect the cognitive function exactly; it is known to sometimes be influenced by the education level of the subject. Improved detection of cognitive impairment among HD patients is required in future studies. Second, this was a cross-sectional study; thus, associations between HD and cognitive impairment suggest, but do not provide evidence for a causal relation. It is necessary to conduct a longitudinal study to clarify the reason for impaired cognition in HD patients. Third, not all possible parameters were included in this study such as dietary habits, atherosclerosis, genetic factors and medications. Further investigation including important confounders is required.

## Conclusions

This report describes the largest study to date assessing the effect of HD on cognitive function and the first report comparing cognitive function between HD patients and the general population in Japan. We found that HD treatment was an independent risk factor for cognitive impairment. In addition, serum sodium level, dry weight and history of cerebrovascular disease tended to associate with MMSE score among HD patients. There is a high prevalence of cognitive impairment among HD patients that has adverse implications for hospitalization and reduced life expectancy. Therefore, cognitive impairment in HD patients should be monitored carefully and treated in an appropriate manner.

## Competing interests

The authors declare that they have no competing interests.

## Authors' contributions

GO conceived the study, designed the study, and wrote the initial draft of the manuscript. NS conducted the statistical analysis, and interpreted the data. SK had full access to all of the data in the study and takes responsibility for the integrity of the data and the accuracy of the data analysis. AK, HS and NYF contributed to study design and assisted in drafting the manuscript. IT and TU participated in the data collection. All authors have approved the manuscript.
